# Regular Black Bean Consumption Is Necessary to Sustain Improvements in Small-Artery Vascular Compliance in the Spontaneously Hypertensive Rat

**DOI:** 10.3390/nu12030685

**Published:** 2020-03-03

**Authors:** Jaime L. Clark, Tara B. Loader, Hope D. Anderson, Peter Zahradka, Carla G. Taylor

**Affiliations:** 1 Department of Food and Human Nutritional Sciences, Faculty of Agriculture and Food Sciences, University of Manitoba, Winnipeg, MB R3T 2N2, Canada; jclark@sbrc.ca (J.L.C.); tloader@sbrc.ca (T.B.L.); pzahradka@sbrc.ca (P.Z.); 2 Canadian Centre for Agri-Food Research in Health and Medicine, St. Boniface Hospital Albrechtsen Research Centre, Winnipeg, MB R2H 2A6, Canada; handerson@sbrc.ca; 3 College of Pharmacy, Rady Faculty of Health Sciences, University of Manitoba, Winnipeg, MB R3E 0T5, Canada; 4 Department of Pharmacology and Therapeutics, Rady Faculty of Health Sciences, University of Manitoba, Winnipeg, MB R3E 0T5, Canada; 5 Department of Physiology and Pathophysiology, Rady Faculty of Health Sciences, University of Manitoba, Winnipeg, MB R3E 0T5, Canada

**Keywords:** black beans, navy beans, hypertension, vascular remodeling, vascular compliance, vascular stiffness, spontaneously hypertensive rat, pressure myography, body composition

## Abstract

Edible legume seeds, such as lentils, have been shown to modulate the structural and functional properties of hypertensive blood vessels, however, the effects of dried beans have not been similarly evaluated. To determine whether beans could attenuate hypertension-induced vascular changes (remodeling and stiffness) in relation to their phytochemical content, spontaneously hypertensive rats (SHR) were fed diets containing black beans (BB; high phytochemical content as indicated by their dark seed coat colour) or navy (white) beans (NB; low phytochemical content) for eight weeks. An additional follow-up phase was included to determine how long the alterations in vascular properties are maintained after bean consumption is halted. Assessments included blood pressure (BP), pulse wave velocity (PWV), vessel compliance (small-artery) and morphology (large-artery), and body composition. Neither BBs nor NBs altered BP or PWV in SHR. SHR-BB demonstrated greater medial strain (which is indicative of greater elasticity) at higher intraluminal pressures (80 and 140 mmHg) compared to SHR-NB. BB consumption for 8 weeks enhanced vascular compliance compared to SHR-NB, as demonstrated by a rightward shift in the stress–strain curve, but this improvement was lost within 2 weeks after halting bean consumption. BB and NB increased lean mass after 8 weeks, but halting BB consumption increased fat mass. In conclusion, regular consumption of BBs may be appropriate as a dietary anti-hypertensive strategy via their positive actions on vascular remodeling and compliance.

## 1. Introduction

Hypertension occurs in more than one in five Canadian adults with 95% of cases having an unknown etiology (therefore known as essential hypertension), thus making treatment difficult [1,2]. Current prescription drug therapy for hypertension focuses on controlling high blood pressure (BP). Unfortunately, for approximately 30% of individuals taking these medications, hypertension remains uncontrolled [2]. This gap in treatment efficacy suggests that it may be beneficial to pursue therapeutic strategies that target the underlying vascular structural changes responsible for hypertension (i.e. vascular remodeling [3]), rather than the clinical symptoms such as elevated systolic blood pressure (SBP) and/or diastolic blood pressure (DBP).

Although the cause of essential hypertension remains unknown, diet is identified as a modifiable risk factor for this condition [4]. A meta-analysis has shown that various pulses (beans, peas, chickpeas, lentils) exert a BP-lowering effect in hypertensive individuals [5]. Furthermore, lentils, consumed whole [6,7] or as a polyphenol-rich extract [8], have been reported to mitigate structural and functional changes in hypertensive blood vessels. Given the similar nutrient contents across the many types of pulse seeds [9], variations in phytochemical composition of these seeds could be responsible for the different therapeutic effects related to vascular function. Phytochemicals are structurally diverse, naturally occurring compounds found in plants [10]. The type of phytochemicals and their biological activity in vivo differs depending on the type, cultivar, and color of the parent plant [11,12]. Among the types of pulse seeds, dried beans have the most variety of types and seed colors [9]. Black beans in particular, with their dark seed coat color, typically have a greater total phenolic content and antioxidant activity than other bean types [13], but to-date they have yet to be examined for their effects on vascular remodeling and compliance. 

The first aim of this study was to compare beans with contrasting seed coat colors (black bean versus white navy beans) for their effects on BP, vascular function, and vascular remodeling after 8 weeks of intervention in a rodent model of essential hypertension. The second aim was to determine if regular consumption of the effective bean is required for maintenance of the vascular and metabolic effects by performing assessments at 2 and 4 weeks during a washout phase. We hypothesized that black beans, with greater phytochemical content as suggested by their dark seed coat color, would attenuate the progression of hypertension and improve blood vessel function and structure in spontaneously hypertensive rats (SHR). Additionally, we hypothesized that the effective bean would need to be consumed regularly to maintain the vascular and metabolic benefits. 

## 2. Materials and Methods 

### 2.1. Animals

Fifteen-week old male SHR and Wistar Kyoto (WKY) rats (Charles River Laboratories, Saint-Constant, QC, Canada) were housed individually in standard plastic-bottomed cages in a room maintained at 25℃ with 40%–60% relative humidity and a 12 h light–12 h dark cycle. The rats were acclimated for one week, and thus were 16 weeks old for baseline assessments. In Phase 1, the rats were randomized into four groups (*n* = 10/group) which received one of the specified diets for 8 weeks: (i) normotensive control WKY rats fed bean-free control diet (WKY-CTRL); (ii) SHR fed bean-free control diet (SHR-CTRL); (iii) SHR fed a black bean diet (SHR-BB); and (iv) SHR fed a navy bean diet (SHR-NB). In Phase 2, the SHR were fed a black bean diet for 8 weeks (*n* = 10) and subsequently were randomized into two washout groups: i) fed bean-free control diet for 2 weeks (*n* = 5) or ii) fed bean-free control diet for 4 weeks (*n* = 5). Black beans were used in Phase 2 since they were considered the more effective bean based on preliminary vascular compliance results obtained from Phase 1. These experiments were carried out in accordance to proper animal care and experimentation as outlined by the Canadian Council on Animal Care and a protocol (15-043) approved on October 7, 2015 by the University of Manitoba Animal Care Committee.

### 2.2. Diet

Diets were formulated and prepared as previously described [6] (Table 1). Black beans (~90% Eclipse, ~10% unknown variety) and navy beans (~75% T9905, ~20% Envoy, ~5% unknown variety) were sourced from Legumex Walker (Plum Coulee, MB). Whole beans were soaked overnight, boiled, freeze-dried, milled to a fine powder using a Retsch ZM 200 Ultra Centrifugal Mill (Retsch, Haan, Germany) at 12,000 rpm with a 0.5 mm screen, and then manually added to the diet and thoroughly incorporated using a commercial floor mixer (Hobart Legacy®, Hobart, Ohio, OH, USA). The bean powders were added to the diet at a 30% *w/w* inclusion which represents approximately 35% of total energy from beans. Diets were provided ad libitum. The previous day’s unconsumed feed was weighed to determine daily feed intake.

### 2.3. Body Weight and Composition

Body weights were measured weekly during both Phases. Whole body composition (fat mass and lean body mass) was assessed in vivo using an EchoMRI-700 whole body quantitative magnetic resonance instrument (EchoMRI, Houston, TX, USA) at baseline and 8 weeks in Phase 1 and at baseline, 8, 10, and 12 weeks in Phase 2. 

### 2.4. Serum Biochemistry

Rats were fasted for 6 hours with free access to water before blood samples were collected from the saphenous vein at baseline and 8 weeks for Phases 1 and 2, with additional collections at 10 and 12 weeks for Phase 2. Serum HDL-C, LDL-C, TC, and TG concentrations were determined using a Cobas c111 auto analyzer (Roche Diagnostics, Indianapolis, IN, USA). Fasting blood collection was performed on separate days from the vascular measurements.

### 2.5. Blood Pressure and Pulse Wave Velocity

BP was measured by tail-cuff plethysmography (CODA system; Kent Scientific, Torrington, CT, USA) at 0, 4, and 8 weeks for Phases 1 and 2, and at 10 and 12 weeks in Phase 2. A minimum of five values were obtained per animal per time point for SBP, DBP and mean arterial pressure (MAP). Pulse wave velocity (PWV) of the femoral artery of anesthetized rats was measured at baseline and 8 weeks for Phases 1 and 2, and at 10 and 12 weeks in Phase 2, with a 10-MHz electrocardiogram-triggered Doppler probe (Indus Instruments, Houston, TX, USA). Three trace measurements were obtained per animal per time point. Analysis of the PWV data was done in a blinded manner using the Doppler Signal Processing Workstation program (DSPW Version 1.624; Indus Instruments, Houston, TX, USA) to determine peak velocity, mean flow velocity and minimum flow velocity. BP and PWV measurements were performed on separate days. 

### 2.6. Tissue Collection

At endpoints for each Phase (8, or 10 and 12 weeks), rats were euthanized by intraperitoneal injection of sodium pentobarbital, followed by decapitation (with rats held horizontally during blood collection to minimize contamination of the blood sample by gastric fluid [15]). The aorta and mesenteric bed were excised for further analyses. The aortae were placed into Optimal Cutting Temperature Compound embedding media (Sakura Finetek USA Inc., Torrence, CA, USA), frozen in a dry ice/ethanol bath, and stored at −80 °C until analyzed.

### 2.7. Pressure Myography of Resistance Arteries

Immediately following dissection, a third order vessel was isolated from the first 10 cm of mesenteric fat and mounted on a pressure myograph (Living Systems Instrumentation, Burlington, VT, USA), pressurized to 45 mmHg, allowed to equilibrate for 1 h in Krebs–Henseleit (KH) buffer (25 mM NaHCO_3_, 5.5 mM glucose, 2.7 μM NaEDTA, 120 mM NaCl, 4.7 mM KCl, 1.2 mM MgSO_4_, 1.2 mM KH_2_PO_4_, 2.5 mM CaCl_2_, pH 7.4) at 37 °C, then challenged with 125 mM KCl solution (in KH buffer) to constrict and confirm arterial viability. After 30 min in Ca^2+^-free KH buffer containing 10 mM egtazic acid (EGTA), triplicate measurements of lumen diameter and left and right wall thickness were recorded at 3, 10, 20, 30, 40, 60, 80, 100, 120, and 140 mmHg.

### 2.8. Aortic Morphology

Five μm sections of aortae were prepared using a Thermo Shandon Cryotome (Thermo Fisher Scientific, Waltham, MA, USA). The slides were kept frozen at −20 °C until stained using an Elastin Stain Kit (Sigma-Aldrich; St. Louis, MO, USA). The slides were fixed in 1% paraformaldehyde for 10 min, washed in 1× phosphate buffered saline (137 mM NaCl, 3 mM KCl, 10 mM Na_2_HPO_4_ and 1.5 mM KH_2_PO_4_) for 10 min, hydrated in double-distilled water for 15 min, and then stained according to the manufacturer’s protocol. Differentiation in the working ferric chloride solution was done for 3 min and staining in the Van Gieson solution was for 90 seconds. Slides were dehydrated in xylene, air-dried for 1 hour, and then mounted using VectaMount Permanent Mounting Medium. Slides were imaged with an EVOS^®^ microscope (Advanced Microscopy Group Solutions) under 4× and 20× objectives. ImageJ software was used to determine vessel morphology (4 × objective), and elastin and collagen content (stained black and red, respectively; 20 × objective) [16]. 

### 2.9. Statistical Analyses

Data were analyzed using repeated-measures ANOVA for time-course data (body weight, body composition, serum analysis, BP, PWV) and one-way ANOVA for endpoint data using Statistical Analysis Software (version 9.3). Duncan’s multiple range post hoc test was used to determine significance among the means. Data that were not normal after log transformation (serum LDL-C, serum TG, body weight, aortic lumen diameter and cross-sectional area (CSA), aortic collagen content (total and relative), and aortic collagen:elastin ratio), as determined by the Shapiro-Wilk normality test, were analyzed using the Wilcoxon Rank–Sum test for non-parametric data followed by Tukey-Kramer multiple comparison test to determine differences between least-squares means. A Box–Cox analysis was used to determine the most appropriate power transformation for nonlinear data (media stress and strain) and this resulted in the square-root transformation being used for media stress. Slope of linear data (total fat vs. lean mass and media stress vs. strain) were calculated for individual animals within each study group using the equation for a line (y = mx + b; where m equals slope), and the slope values were used for statistical analysis. Results were considered statistically significant at *p* < 0.05.

## 3. Results

### 3.1. Phase 1: Comparing the Effects of Navy and Black Beans

#### 3.1.1. Feed Intake and Body Composition

**Figure 1:**
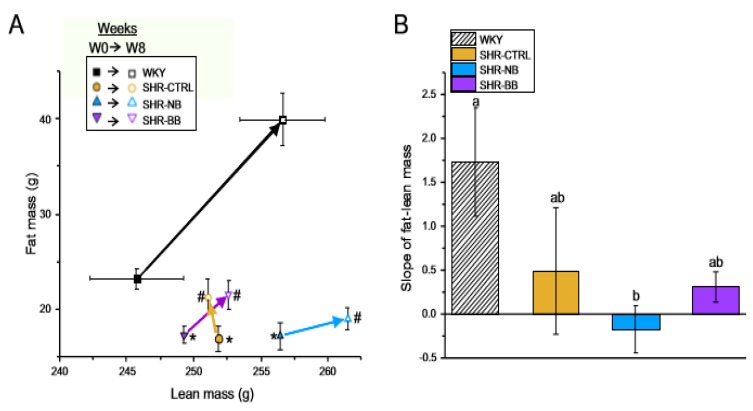
Phase 1: Whole body fat mass and lean mass. (**A**) Whole body fat mass-lean mass relationship at baseline compared to week 8; (**B**) Slope of the whole body fat mass-lean mass relationship. Measurements of fat mass and lean mass were obtained in vivo using an EchoMRI whole body quantitative magnetic resonance instrument and plotted with fat mass on the Y axis and lean mass on the X axis with closed symbols for baseline and open symbols for week 8. In Panel A, arrows between time points indicate direction of change from baseline to week 8. Data are expressed as mean ± SEM (*n* = 9–10/group); SEM bars for lean mass are too small to be visible in the figure. *, fat mass significantly different (*p* < 0.05) compared to WKY at baseline; #, fat mass significantly different (*p* < 0.05) compared to WKY at week 8. In Panel B, columns with different letters are significantly different (*p* < 0.05). Abbreviations: BB, black bean diet; CTRL, bean-free control diet; NB, navy bean diet; SHR, spontaneously hypertensive rat; W0, Week 0 (baseline); W8, Week 8; WKY, Wistar Kyoto.

During week 8, SHR consumed more feed but had a lower body weight compared to WKY rats, regardless of the dietary intervention (Table 2). There was a distinct separation in whole body fat mass and lean mass between WKY and SHR animals at baseline and week 8 (Figure 1a). The rightward direction of the linear relationship (positive slope) between fat mass and lean mass indicates an increase in lean mass over time, whereas the upward direction indicates an increase in fat mass over time. The WKY rats had the greatest increase in fat mass and lean mass over 8 weeks, and this slope was significantly different from the slope of the SHR consuming the navy bean diet (Figure 1b). Interestingly, the relationship between fat mass and lean mass shifted rightward for SHR-NB (presenting as a negative slope for the mean of the group), indicating an increase in lean mass, but not fat mass, over time. There were no differences in the slope of the fat–lean mass relationship among WKY, SHR-CTRL, and SHR-BB groups. 

#### 3.1.2. Serum Lipid Analysis

At baseline and week 8, WKY rats had higher serum TG, TC, HDL-C, and LDL-C than SHR (Table 2), as previously reported [6,7,17]. There were no differences in serum lipids among SHR groups after 8 weeks of bean consumption.

#### 3.1.3. Blood Pressure and Arterial Stiffness

At baseline, week 4 and week 8, all SHR had higher SBP, DBP, and MAP compared to WKY rats (Table 3). There were no diet-related differences in BP among the SHR groups. There were also no differences for heart rate and heart rate variability among the groups. Likewise, PWV measurements (peak flow velocity, minimum flow velocity, mean flow velocity, pulsatility index, and resistivity index) did not differ between any groups, including SHR vs. WKY, at week 8. 

#### 3.1.4. Vascular Remodeling and Geometry

**Figure 2:**
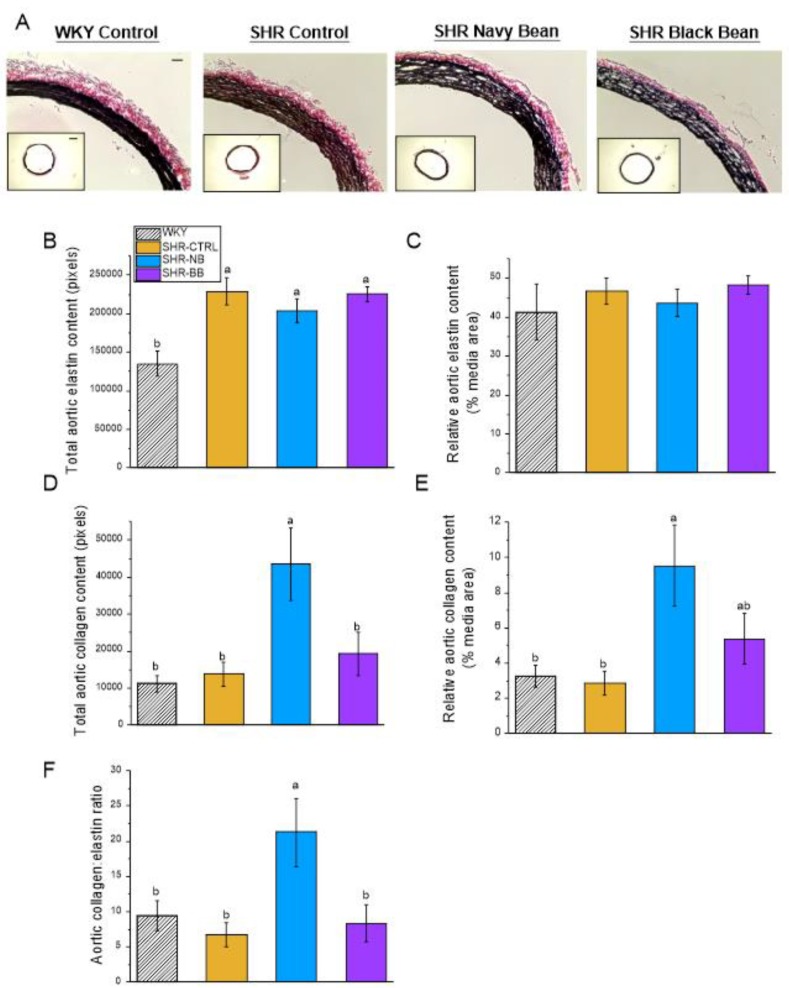
Phase 1: Large-artery elastin and collagen. (**A**) Representative cross-sections (20× objective) of aorta stained for elastin (black) and collagen (red); (**B**) Elastin content in aorta as total area; (**C**) Elastin content in aorta as % media area; (**D**) Collagen content in aorta as total area; (**E**) Collagen content in aorta as % media area; (**F**) Aortic collagen:elastin ratio. Aorta cross-sections were stained with an Elastin Stain Kit (Sigma); elastin and collagen contents were determined using ImageJ to quantify the area of blackness (elastin) and redness (collagen). Inset: aortic cross-sections (4× objective). Scale bar applies equally to all aorta images (bar = 50 µm). Data are expressed as mean ± SEM (*n* = 6–9/group). Different letters represent significant differences (*p* < 0.05). An absence of letters indicates no statistical differences. Abbreviations: BB, black bean diet; CTRL, bean-free control diet; NB, navy bean diet; SHR, spontaneously hypertensive rat; WKY, Wistar Kyoto.

Morphometric analyses of aortic sections revealed no differences among any of the groups with respect to aortic lumen diameter, lumen cross-sectional area (CSA), external diameter, adventitia thickness, or adventitia CSA (Table 4). The WKY group had a smaller aortic media wall thickness, media CSA, and media:lumen ratio than all the SHR groups. Representative sections of aorta stained for elastin and collagen are shown in Figure 2a. There were no differences in total aortic elastin content or elastin content relative to media area between the groups (Figure 2b,c). SHR-NB had greater aortic collagen content (total and relative to media area) compared to WKY and SHR-CTRL. SHR-NB had greater total aortic collagen compared to SHR-BB, but there was no difference for aortic collagen relative to media area (Figure 2d,e). SHR-NB also had a greater collagen:elastin ratio compared to WKY, SHR-CTRL, and SHR-BB (Figure 2f). 

Wall thickness and lumen measurements (vascular geometry) were obtained via myography at a constant intraluminal pressure of 45 mmHg in mesenteric resistance arteries perfused with a Ca^2+^-free KH solution to deactivate myogenic tone [18]. The WKY group had a larger lumen diameter, smaller media thickness and media:lumen ratio, larger external diameter, and similar media CSA compared to SHR (Table 4). There were no diet-related differences in vascular geometry measurements of mesenteric arteries among the SHR groups.

#### 3.1.5. Vascular Compliance

**Figure 3:**
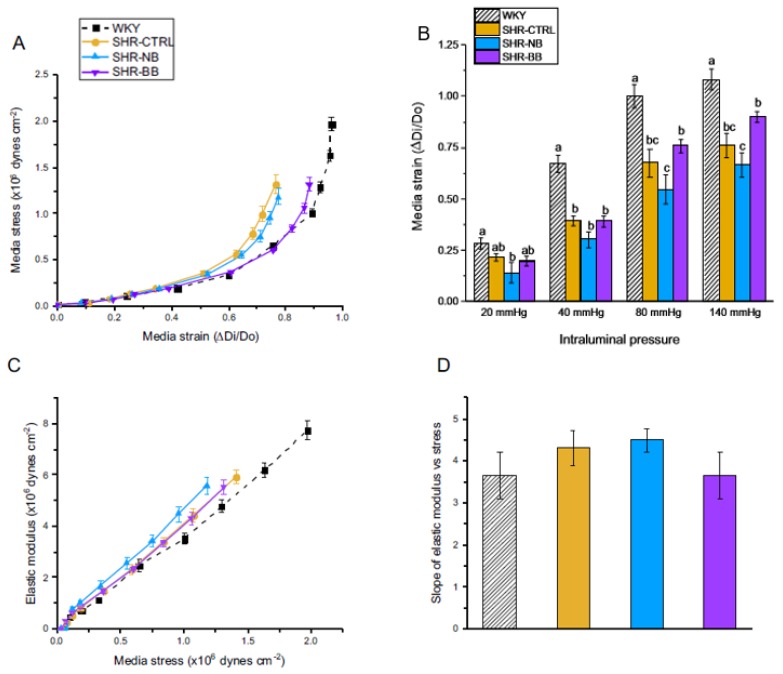
Phase 1: Small-artery vascular compliance. (**A**) Media stress–strain relationship; (**B**) Media strain at intraluminal pressures of 20, 40, 80, and 140 mmHg; (**C**) Elastic modulus vs. media stress; (**D**) Slope of elastic modulus vs. media stress. Vascular compliance measurements of mesenteric resistance arteries were calculated from data obtained at 3, 10, 20, 30, 40, 60, 80, 100, 120, and 140 mmHg on the pressure myograph. Data are expressed as mean ± SEM (*n* = 7–10/group). Different letters represent significant differences (*p* < 0.05) within a given intraluminal pressure in (**B**). An absence of letters indicates no statistical differences in (**D**). Abbreviations: BB, black bean diet; BW, body weight; CTRL, bean-free control diet; NB, navy bean diet; SHR, spontaneously hypertensive rat; WKY, Wistar Kyoto.

Vascular compliance, the ability of a vessel to buffer changes in pressure [19], was measured by plotting the relationship between media stress and media strain [20] for the mesenteric resistance arteries. There was a distinct separation of the stress–strain curves among the four animal groups. SHR-CTRL and SHR-NB show a leftward shift, indicating reduced vascular compliance, while SHR-BB and WKY rats are shifted towards the right, indicating better vascular compliance (Figure 3a). Additionally, SHR-BB demonstrated greater medial strain (which is indicative of greater elasticity) at higher intraluminal pressures (80 and 140 mmHg) compared to SHR-NB (Figure 3b). 

Unlike vascular compliance, incremental elastic modulus depends upon the stiffness of the vessel wall components independent of vessel geometry [19]. When incremental elastic modulus is plotted against media stress, the slope of elastic modulus vs. stress indicates the stiffness of wall components such as elastin, collagen, and SMC [20]. There were no differences in the slope of the elastic modulus vs. media stress among the four groups (Figure 3c,d), despite there being differences in arterial compliance.

### 3.2. Phase 2: Determining the Retention of Beneficial Effects from Black Beans During a Washout Period 

#### 3.2.1. Feed Intake and Body Composition

**Figure 4:**
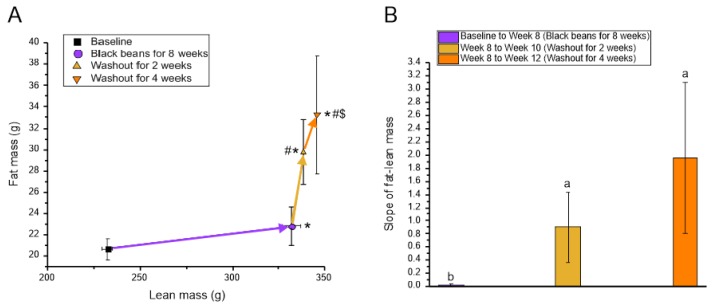
Phase 2: Whole body fat mass and lean mass of SHR. (**A**) Whole body fat-lean mass relationships from baseline to week 8, week 8 to week 10, and week 10 to week 12; (**B**) Slope of whole body fat-lean mass relationships. Measurements were obtained in vivo using an EchoMRI whole body quantitative magnetic resonance instrument. In Panel A, arrows between time points indicate direction of change. Data are expressed as mean ± SEM (*n* = 4–9/group); some SEM bars for lean mass are too small to be visible in the figure. *, lean mass significantly different (*p* < 0.05) from baseline; **#**, fat mass significantly different (*p* < 0.05) from baseline; **$**, fat mass significantly different (*p* < 0.05) from week 8. In Panel B, different letters represent significant differences (*p* < 0.05). Abbreviations: SHR, spontaneously hypertensive rats.

SHR fed the black bean diet had an increased daily feed intake at 8 weeks compared to baseline, but there were no differences in daily feed intake between 8 and 12 weeks when they consumed the bean-free diet (Table 5). Similar to Phase 1, SHR gained weight during the 8 weeks of black bean feeding. There was a further increase in body weight gain during the 4 weeks when SHR were switched from the black bean diet to the bean-free control diet. Whole body fat mass was plotted relative to whole body lean mass (Figure 4a). The rightward direction of the linear relationship (positive slope) between fat mass and lean mass indicates an increase in lean mass over time, as was observed between baseline and 8 weeks of bean consumption. The upward direction indicates an increase in fat mass over time. Whole body fat mass did not change during the 8 weeks of black bean feeding; however, after 2 weeks of a bean-free diet, fat mass was increased compared to baseline. Additionally, after 4 weeks of a bean-free diet, fat mass was increased compared to baseline and 8 weeks of black bean feeding. There was no difference in fat mass between the two washout groups. The slopes of the fat-lean mass relationship were greater for the two washout phases compared to week 8 due to the increase in fat mass, but not lean mass, after bean consumption was discontinued (Figure 4b). 

#### 3.2.2. Serum Lipid Analysis

Consumption of the black bean diet for 8 weeks decreased serum TG by 34% and TC by 16% compared to baseline (Table 5). TG and TC were unchanged during the washout phase. HDL-C remained constant during this experiment whereas LDL-C was unchanged during the washout period compared to the 8-week bean intervention.

#### 3.2.3. Blood Pressure and Arterial Stiffness

SBP, DBP, and MAP did not change after 8 weeks of black bean feeding, nor did they change during the 4-week washout period (Table 6). Heart rate was increased compared to baseline with 8 weeks of black bean feeding, and this increase was maintained during the washout periods. In contrast, heart rate variability was reduced with black bean feeding and this reduction was maintained during the washouts, with no differences between the 2- and 4-week washout periods. Measurements of arterial stiffness (PWV), as well as minimum and mean flow velocity, were reduced after black bean consumption for 8 weeks and remained reduced during the washout periods. Peak flow velocity decreased over time but was not different between the black bean phase and washout phase. Pulsatility index increased following black bean consumption, but was decreased during the washout periods, with no difference between the washout time-points. Resistivity index also increased following 8 weeks of black bean consumption, but it did not significantly decline during the washout periods.

#### 3.2.4. Vascular Geometry

**Figure 5:**
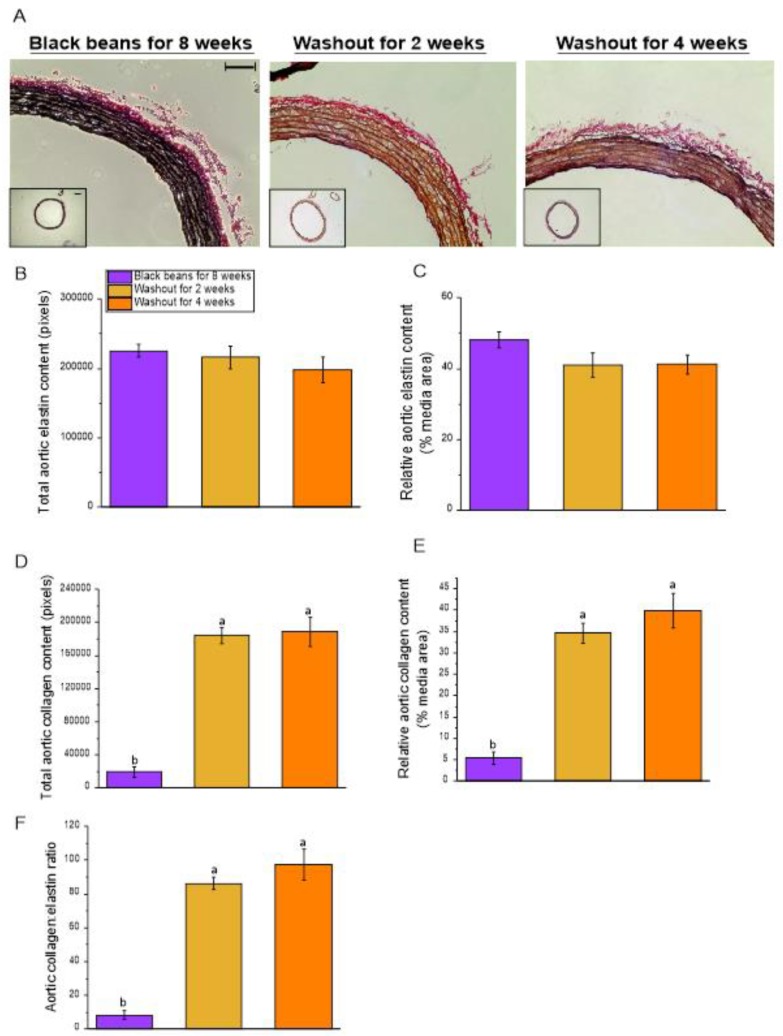
Phase 2: Large-artery elastin and collagen in SHR. (**A**) Representative cross-sections (20× objective) of aorta stained for elastin (black) and collagen (red); (**B**) Elastin content in aorta as total area; (**C**) Elastin content in aorta as % media area; (**D**) Collagen content in aorta as total area; (**E**) Collagen content in aorta as % media area; (**F**) Aortic collagen:elastin ratio. Aortic cross-sections were stained with an Elastin Stain Kit (Sigma); ImageJ was used to quantify elastin (area of blackness) and collagen (area of redness) content. Inset: aortic cross-sections (4× objective). Scale bar applies equally to all aorta images (bar = 50 µm). Data are expressed as mean ± SEM (*n* = 4–7/group). Different letters represent significant differences (*p* < 0.05). An absence of letters indicates no statistical differences. Abbreviations: SHR, spontaneously hypertensive rat.

Morphometric analyses of aortic sections revealed no differences among SHR fed black beans for 8 weeks and the two washout groups with respect to aortic lumen diameter, lumen CSA, external diameter, adventitia thickness, and adventitia CSA (Table 7). Media thickness and CSA were increased in the two washout groups compared to the black bean group at 8 weeks. Media:lumen ratio was increased after four weeks of washout diet but was not different between 8 weeks of black bean feeding and 2 weeks of washout diet. Aortic sections stained for elastin and collagen (Figure 5a) showed no differences in total aortic elastin content or elastin content relative to media area among SHR fed black beans for 8 weeks and the two washout groups (Figure 5b,c). Total aortic collagen content, collagen content relative to media area (Figure 5d,e), and collagen:elastin ratio (Figure 5f) were higher in the two washout groups compared to the black bean group.

**Figure 6:**
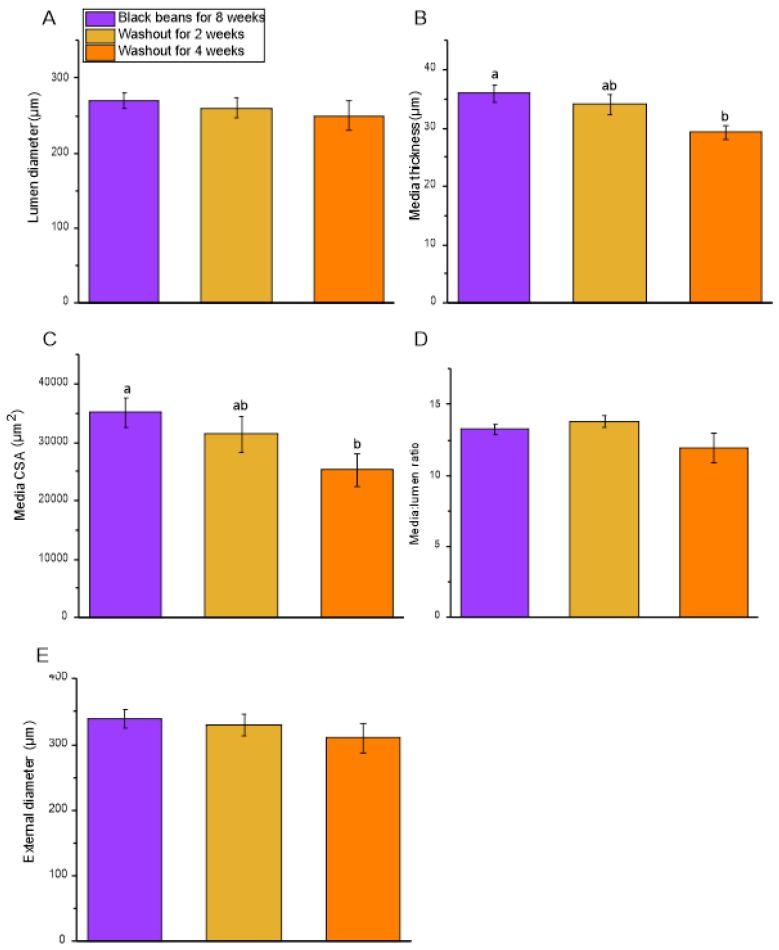
Phase 2: Small-artery geometry in SHR. (**A**) Lumen diameter; (**B**) Media thickness; (**C**) Media CSA; (**D**) Media:lumen ratio; (**E**) External diameter. Vascular geometry measurements of mesenteric resistance arteries calculated from data obtained at 45 mmHg on the pressure myograph. Data are expressed as mean ± SEM (*n* = 4–9/group). Different letters represent significant differences (*p* < 0.05). An absence of letters indicates no statistical differences. Abbreviations: CSA, cross-sectional area; SHR, spontaneously hypertensive rat.

There was a significant effect of the bean washout period on media thickness and media CSA of the mesenteric resistance arteries (Figure 6). Media thickness and CSA began to decrease within two weeks on the bean-free control diet, and after four weeks on the washout diet, these parameters were different from the rats which had consumed black beans for 8 weeks. Lumen diameter, external diameter, and media:lumen ratio were unchanged.

#### 3.2.5. Vascular Compliance

**Figure 7:**
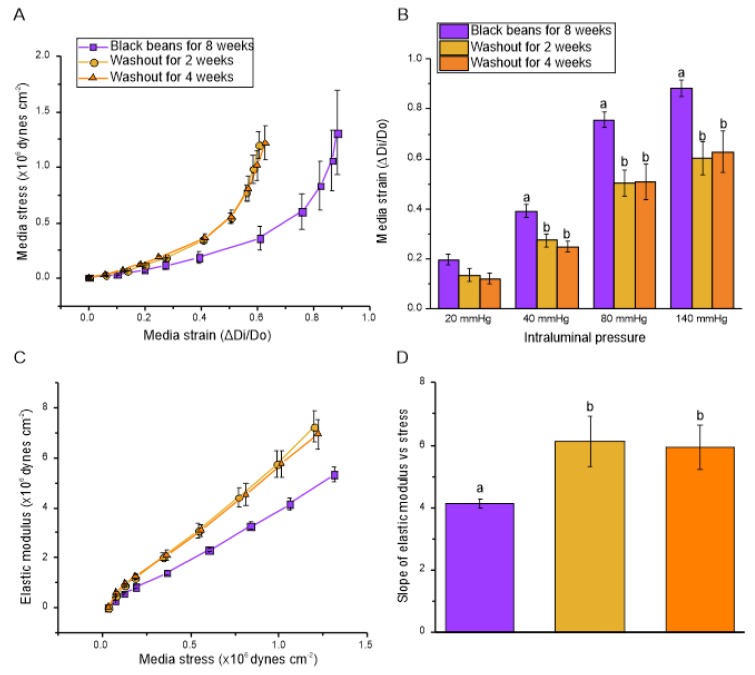
Phase 2: Small-artery vascular compliance in SHR: (**A**) Media stress–strain relationship; (**B**) Media strain at intraluminal pressures of 20, 40, 80, and 140 mmHg; (**C**) Elastic modulus vs. media stress; (**D**) Slope of elastic modulus vs. media stress. Vascular compliance measurements of mesenteric resistance arteries were calculated from data obtained at 3, 10, 20, 30, 40, 60, 80, 100, 120, and 140 mmHg on the pressure myograph. Data are expressed as mean ± SEM (*n* = 4–6/group). Different letters represent significant differences (*p* < 0.05) within a given intraluminal pressure in (B). An absence of letters indicates no statistical differences in (D). Abbreviation: SHR, spontaneously hypertensive rat.

The leftward shift of the stress–strain curve in mesenteric resistance arteries of the SHR no longer consuming black beans for 2 or 4 weeks indicates a loss of vascular compliance compared to the SHR after 8 weeks of black bean consumption (Figure 7a). In concurrence with this observation, SHR that underwent the washout phase had a lower medial strain at intraluminal pressures of 40, 80, and 140 mmHg compared to the SHR rats that continuously ate black beans (Figure 7b).

The switch to a bean-free diet after 8-weeks of continuous black bean feeding increased the slope of the elastic modulus vs. circumferential stress in SHR (Figure 7c,d), indicating an increase in wall component stiffness. There were no differences between the two washout periods for either assessment of vascular compliance. 

## 4. Discussion

The principal findings of this study established that consumption of black beans, but not navy beans: (i) improved vascular compliance, based on a rightward shift of the stress–strain curve; (ii) resulted in greater medial strain (elasticity) when arteries were exposed to higher intraluminal pressures; and (iii) partially attenuated vascular remodeling in SHR. We also investigated whether the vascular effects elicited by black beans persist once their consumption has stopped. This produced the novel finding that improvements in vascular function, as determined by arterial compliance and stiffness, and reductions serum LDL-C levels, were not sustained when black bean consumption had ceased during the 2- and 4-week washout periods. Additionally, we observed that both black and navy beans preferentially enhanced lean mass accumulation at the expense of fat tissue, and that the opposite occurred when bean consumption ceased. The results from this research indicate that regular long-term consumption of black beans is necessary to achieve and maintain the vascular and metabolic benefits associated with their consumption. 

In this study, we investigated the effects of a high dose of beans (30% *w/w*), which contributed 35% total energy intake for SHR, on various metabolic and vascular parameters. This was a higher dose than is typical for a western country such as Canada, as Mudryj et al. [21] have reported that the highest quartile of pulse consumers in Canada ate >137.2 g pulses/day, representing approximately 19% of their daily caloric intake. Future studies investigating the physiological effects of lower bean doses (for example, 7.5% or 15% *w/w*) in SHR would be more applicable to human intakes in developed countries. 

In the present study, SHR fed black beans, but not navy beans, demonstrated improved compliance of resistance arteries (<400 µm luminal diameter; located in the mesenteric adipose depot) compared to WKY rats fed control diet, as depicted by the rightward shift of the stress–strain curve and the subsequent smaller slope change of the stress–strain curve [22,23]. This shift of the stress–strain curve is most likely due to the retention of elastic fiber engagement or lower engagement of collagen in the vessel wall, both being associated with preserved arterial compliance under conditions of increasing pressure [24]. As strain (stretch) is applied with increasing intraluminal pressure, collagen fibers become engaged within the vessel wall to preserve the shape of the tissue, thereby increasing vessel wall stiffness [24]. Navy bean consumption demonstrated lower medial strain (less stretch) at higher intraluminal pressures compared to black beans. This finding potentially indicates an earlier loss of elasticity or earlier increase in stiffness (collagen) within the vessel wall of SHR fed navy beans, compared to that of SHR fed black beans. 

There were no changes in wall component stiffness (elastic modulus vs. stress) or small-artery geometry in response to black bean feeding, both of which are factors that determine vascular compliance [25]. There is no ready explanation as to why vascular compliance was changed, but not its determinable factors. However, eating black beans resulted in less total collagen content in the aorta, an elastic artery with a significantly larger diameter than the mesenteric vessels, and a reduction in the collagen: elastin ratio compared to navy beans. The changes in collagen content in the aorta of the bean-fed SHR may also be reflected in the smaller mesenteric resistance arteries. Such differences in vascular collagen content between navy bean- and black bean-fed SHR may account for the differences in vascular strain and compliance. On the other hand, black bean consumption by SHR had no effect on elastic artery geometry compared to navy beans or the bean-free control diet, despite improvements in the compliance of resistant arteries. A similar finding occurred in SHR fed green lentils for 8 weeks [7]. Since smaller resistance arteries have a greater proportion of smooth muscle (relative to vessel size) compared to large elastic arteries like the aorta [26], the positive effects of black beans in smaller arteries could be due to a greater change in vascular smooth muscle cell (VSMC) activity within the mesenteric arterial bed. Although it is currently unknown whether bioactive compounds from black beans have selective actions within the vasculature, further exploration could involve investigating cultured SMCs to screen for bioactive compounds that promote the contractile phenotype or, alternatively, compounds that prevent the switch from the contractile to the synthetic state. 

Navy beans exhibited negative effects on small and large arteries as evidenced by reduced vascular compliance and medial strain of small resistance arteries and increased aortic collagen content compared to WKY rats and SHR fed black beans. There is no ready explanation for the increased aortic collagen content of SHR fed navy beans. However, if the increased collagen content is reflective of increased collagen synthesis, this could indicate navy beans are promoting the shift of VSMC to the synthetic phenotype [27]. Further investigation is required to confirm this possibility. This is the first study to report negative findings in relation to navy bean consumption. Interestingly, Monk et al. [28] reported that a diet containing black beans enhanced colonic barrier integrity and function as well as the microbiotic composition in mice compared to those fed navy beans, and they attributed the improvements in gut health to the unique components of black beans, particularly their polyphenols. It is likely that the phytochemical differences between the two bean types are also responsible for their different vascular effects, given that the nutritional composition of black beans and navy beans is similar (Table S1). It is currently unknown if navy beans impair or are just ineffective for improving hypertension-induced structural and functional changes. Studies have shown navy beans to be beneficial for other disease states, such colon cancer [29,30] and obesity [31]. Therefore, it is possible that the benefits of navy beans are not preferential to the vasculature but to other metabolic tissues, which may be due to target specificity of their polyphenolic compounds. The distinct biological effects of navy beans and black beans during different disease states may support the inclusion of both beans in the diet for optimal health effects. However, it is important to note that our study did not investigate a mixed diet of black and navy beans, therefore, it is unknown whether or not the vascular benefits of black beans would be blunted by navy beans. Overall, our results indicate that black beans are the optimal choice for improving vascular function, but further investigation is required to determine if navy beans have a negative effect on the vasculature. 

Interestingly, Phase 2 demonstrated changes in both small- and large-artery geometry upon cessation of black bean intake. However, the small mesenteric arteries showed decreased media thickness and CSA, without changes in lumen diameter, whereas aortae showed increases in media thickness, CSA, and media: lumen ratio, without changes in lumen diameter, after 4 weeks of bean-free diet compared to 8 weeks of black bean feeding. The differences in media geometry between the two artery types suggest local differences in VSMC activity, phenotype modulation, and/or remodeling (e.g., elastin, collagen). The increase in aortic media thickness and area in SHR after the washout phases are likely due to the higher amounts of collagen present in the aorta. 

Phase 2 of our study has provided novel information regarding the retention of the biological activity associated with black beans. Improvements to vascular compliance of mesenteric resistance arteries induced by 8 weeks of black bean consumption were lost after halting black bean consumption for 2 weeks. As well, vessel elasticity at higher intraluminal pressures was diminished within 2 weeks after black bean intake. This finding suggests that in the absence of black beans, the mechanical properties of the blood vessels shift wherein the vessels do not retain their ability to stretch with increasing pressure loads. Overall, it would appear that the bioactive compounds obtained from long-term consumption of black beans, which are the likely effectors for the vascular improvements, are not sufficiently retained within the tissues, which would explain why their effects last for less than 2 weeks. Therefore, it is likely that with regular consumption of black beans these effector compounds could be maintained within the vascular tissue and continue to promote their beneficial effects on the vasculature. Indeed, retention of bioactive compounds, such as polyphenols, has not been measured in vascular tissues, and therefore the kinetics of phytochemical penetration and elimination at the tissue and cellular level is unknown [32]. Alternatively, the changes in endogenous metabolites that promote better vascular functionality upon the consumption of black beans may be swiftly lost once black beans are no longer consumed. This would be the case if the vascular changes occur because the bioactive compounds in black beans target the underlying cause of those changes. Thus, the absence of the compounds in the bean-free control diet would allow the pathophysiological processes to again exert their negative effects on the vessels. It was novel, yet unsurprising, that the positive effects on small artery function were reversed following the cessation of black bean consumption. VSMCs are dynamic in nature, allowing for the immediate responses required to regulate BP and blood flow [33]. They are also susceptible to phenotype switching, wherein they can become proliferative and promote vascular dysfunction [34,35]. Without the continual intake of black beans and their bioactive compounds, it is likely the endogenous pathways that attenuate small-artery stiffness under the high BP conditions of the SHR circulatory system are no longer operational. Thus, VSMC adapt to the new vascular environment which favors stiffness. 

Anthocyanins are the most probable phytochemical compounds responsible for the beneficial effects on the vasculature in our study simply because they are present in black beans and not in white beans [36,37]. Additionally, anthocyanins, as constituents of whole grape powder, were speculated to be responsible for improving arterial compliance in mesenteric resistance arteries of SHR [25]. The seed coats of black bean also contain flavonols such as myricetin, quercetin, and kaempferol glycosides, which are not present in the seed coats of navy (white) beans [38]. Dry beans also contain isoflavone compounds that are present at higher amounts in black beans than lighter-colored beans [39]. Nestel et al. [40] reported that arterial compliance was higher in menopausal women after supplementation with soy isoflavone for five weeks compared to the placebo group. While flavonoids (e.g., anthocyanins and flavonols) and isoflavones derived from beans have not been directly investigated for their vascular benefits, the literature does indicate substantial differences in phytochemical compounds between navy beans and black beans [36,37,38,39], and these compounds are the likely effectors for the differing vascular effects observed in the present study. 

There was a positive effect of navy beans, but not black beans, on body composition in SHR during Phase 1. Navy beans increased lean mass after 8 weeks, which is considered a favorable direction for changes in body composition [41]. Maintaining and increasing lean mass is important since skeletal muscle and bone loss (both components of lean mass) occur with age and increase the risk for osteoporosis, falls, fractures, physical disabilities, and mortality [42,43]. Thus, the ability of beans to prevent lean mass loss over 8 weeks could be beneficial for preventing age-related declines in muscle and bone mass. It is important to note that the current study did not measure bone or muscle mass separate of total lean mass, therefore, it cannot be determined if the beans act directly on these individual components. Interestingly, black beans did not elicit this effect on body composition in Phase 1 but did in Phase 2. There is no ready explanation for the discrepancy in the effects of black beans on body composition between the two phases. Overall, the ability of light- and dark-colored beans to positively affect both whole body lean mass and fat mass may add to the evidence supporting dietary pulses as part of a healthy diet for body weight loss and maintenance since decreasing fat and increasing or preserving muscle mass is recommended as part of a healthy weight loss strategy [44]. Thus, while our data suggest beans may be beneficial for preventing age-related muscle and bone loss, further investigation is required to validate this observation.

As previously mentioned, favorable metabolic changes occurred in response to black bean feeding in Phase 2, where black beans increased lean mass without changing fat mass after 8 weeks. Interestingly, when the consumption of black beans ceased, the increase in lean mass stalled. At the same time, both fat mass and LDL-C began to increase. The loss of these positive metabolic effects suggests that black beans need to be continually consumed to maintain their beneficial effects on body composition and LDL-C. 

It bears mentioning that the design of Phase 2 did not include age-matched control animals to properly discern the effects of the bean washout from that of aging. However, based on the literature, the vascular changes observed by the 25–29 weeks old SHR in the current study are most likely due to the change in diet, and are not related to age. Chen et al. [45] noted that blood pressure and the left ventricle weight of SHRs plateaus between 16 and 40 weeks old. The progression to left ventricular hypertrophy is usually preceded by structural changes to resistance and large arteries [46]. Therefore, it would be expected that the plateau in blood pressure and left ventricular weight would also be accompanied by a plateau for other vascular properties, such as compliance and stiffness. 

## 5. Conclusions

In conclusion, this study explored the comparative effects of beans with contrasting seed coat colors (black vs. navy beans) on vascular and metabolic parameters in SHR, as a model of essential hypertension. The unique results of this study suggest that not only could black beans be used as part of a potential therapeutic dietary strategy to combat the remodeling and diminished compliance of blood vessels caused by hypertension, but also that adhering to regular consumption of black beans, without discontinuation, is necessary to maintain their vascular benefits. Furthermore, both bean types proved effective in maintaining whole body lean mass and attenuating fat mass during aging—an effect which was reversed upon cessation of black beans. Overall, the results of this study suggest that regular inclusion of black beans in the diet may be beneficial for improving vascular health and attenuating progressive damage from hypertension, but also for maintaining whole body lean mass during aging. Further investigation is warranted to identify both the mechanism(s) behind the observed vascular and metabolic improvements and the factor(s) responsible for them.

## Figures and Tables

**Table 1:** Experimental diet formulations.

	Bean-Free Control Diet ^1^	Navy Bean Diet	Black Bean Diet
**Ingredients ^2,3^ (g/kg)**			
Casein	200	118	121.5
Maize starch	397	229.5	226
Maltodextrin	132	132	132
Sucrose	100	100	100
Cellulose	50	0	50
L-Cystine	3	3	3
Choline bitartrate	2.5	2.5	2.5
AIN-93G-MX mineral mix	35	35	35
AIN-93-VX vitamin mix	10	10	10
Soybean oil ^4^	70	70	70
**Pulse powders ^5^ (g/kg)**			
Navy bean	-	300	-
Black bean	-	-	300

^1^ American Institute of Nutrition-93G formulation [14]. ^2^ Ingredients (except pulse powders) from Dyets, Inc. (Bethlehem, PA, USA). ^3^ Adjustments to casein, maize starch, and cellulose for pulse diets were based on proximate analysis of the pulse powders (see Table S1). ^4^ With 0.02% tert-butylhydroquinone. ^5^ Pulse powders were prepared as described in Methods.

**Table 2:** Phase 1: Metabolic parameters.

	WKY	SHR		
	Control	Control	Navy Bean	Black Bean
**Feed intake (g/day)** Week 1 Week 8	17.6 ± 0.6 24.8 ± 2.4 ^b^	20.9 ± 1.5 43.5 ± 2.7 ^a^	18.4 ± 1.8 34.9 ± 4.1 ^a^	21.3 ± 1.7 39.2 ± 4.5 ^a^
**Body weight (g)** Baseline Week 8	336 ± 6 407 ± 8 ^a^	324± 6 370 ± 5 ^b^	338 ± 3 379 ± 5 ^b^	335 ± 4 373 ± 6 ^b^
**Triglycerides (mmol/L)** Baseline Week 8	1.54 ± 0.17 ^a^ 1.41 ± 0.10 ^a^	0.58 ± 0.10 ^b^ 0.59 ± 0.06 ^b^	0.64 ± 0.05 ^b^ 0.69 ± 0.09 ^b^	0.60 ± 0.08 ^b^ 0.62 ± 0.07 ^b^
**Total cholesterol (mmol/L)** Baseline Week 8	2.64 ± 0.09 ^a^ 3.51 ± 0.09 ^a^	1.63 ± 0.05 ^b^ 1.68 ± 0.08 ^b^	1.69 ± 0.05 ^b^ 1.58 ± 0.06 ^b^	1.63 ± 0.03 ^b^ 1.50 ± 0.04 ^b^
**HDL-cholesterol (mmol/L)** Baseline Week 8	2.07 ± 0.08 ^a^ 2.57 ± 0.06 ^a^	1.34 ± 0.03 ^b^ 1.24 ± 0.06 ^b^	1.33 ± 0.04 ^b^ 1.40 ± 0.24 ^b^	1.31 ± 0.03 ^b^ 1.15 ± 0.04 ^b^
**LDL-cholesterol (mmol/L)** Baseline Week 8	0.40 ±0.03 ^a^ 0.77 ± 0.05 ^a^	0.35 ± 0.03 ^a,b^ 0.40 ± 0.04 ^b^	0.30 ± 0.02 ^b^ 0.32 ± 0.04 ^b^	0.30 ± 0.03 ^b^ 0.32 ± 0.01 ^b^

Data are expressed as mean ± SEM (*n* = 8–10/group). For means within the same row, different letters superscripts [a,b] represent significant differences (*p* < 0.05). An absence of letters indicates no statistical differences. Abbreviations: HDL, high-density lipoprotein; LDL, low-density lipoprotein; SHR, spontaneously hypertensive rat; WKY, Wistar Kyoto.

**Table 3:** Phase 1: Hemodynamic properties and arterial stiffness.

	WKY	SHR		
	Control	Control	Navy Bean	Black Bean
**Hemodynamics**				
**SBP (mmHg) ^1^**				
Baseline	153 ± 7 ^b^	197 ± 6 ^a^	181 ± 8 ^a^	187 ± 4 ^a^
Week 4	153 ± 4 ^b^	188 ± 5 ^a^	194 ± 7 ^a^	194 ± 5 ^a^
Week 8	142 ± 6 ^b^	184 ± 5 ^a^	199 ± 6 ^a^	199 ± 6 ^a^
**DBP (mmHg) ^1^**				
Baseline	99 ± 4 ^b^	149 ± 7 ^a^	134 ± 8 ^a^	140 ± 5 ^a^
Week 4	103 ± 5 ^b^	140 ± 4 ^a^	145 ± 7 ^a^	142 ± 7 ^a^
Week 8	94 ± 4 ^b^	138 ± 4 ^a^	146 ± 9 ^a^	144 ± 7 ^a^
**MAP (mmHg) ^1^**				
Baseline	116 ± 5 ^b^	165 ± 6 ^a^	149 ± 8 ^a^	155 ± 5 ^a^
Week 4	119 ± 4 ^b^	156 ± 4 ^a^	161 ± 7 ^a^	159 ± 6 ^a^
Week 8	110 ± 5 ^b^	153 ± 4 ^a^	163 ± 8 ^a^	162 ± 6 ^a^
**HR (bpm) ^2^**				
Baseline	397 ± 7	436 ± 20	435 ± 20	375 ± 7
Week 8	387 ± 14	442 ± 22	423 ± 22	423 ± 16
**HRV (bpm) ^2^**				
Baseline	153 ± 2	145 ± 5	147 ± 4	162 ± 3
Week 8	158 ± 4	145 ± 6	148 ± 5	147 ± 3
**Femoral artery stiffness**				
**PFV (cm/s)**				
Baseline	26.4 ± 1.8	28.3 ± 0.6	27.6 ± 1.1	27.6 ± 0.9
Week 8	24.6 ± 1.3	27.9 ± 1.1	27.3 ± 1.9	27.7 ± 1.4
**MinFV (cm/s)**				
Baseline	1.60 ± 0.78	1.90 ± 1.10	3.32 ± 0.35	2.98 ± 0.54
Week 8	2.74 ± 0.32	3.25 ± 0.73	2.00 ± 0.76	3.85 ± 0.59
**MFV (cm/s)**				
Baseline	6.37 ± 0.59 ^b^	7.44 ± 0.88 ^a,b^	8.55 ± 0.44 ^a^	8.67 ± 0.48 ^a^
Week 8	6.85 ± 0.45	8.20 ± 0.79	7.30 ± 0.56	8.69 ± 0.72
**Pulsatility index**				
Baseline	4.2 ± 0.4	3.7 ± 0.6	2.9 ± 0.1	3.1 ± 0.3
Week 8	2.7 ± 0.4	3.0 ± 0.3	3.3 ± 0.2	3.2 ± 0.3
**Resistivity index**				
Baseline	0.94 ± 0.03	0.92 ± 0.03	0.88 ± 0.01	0.90 ± 0.02
Week 8	0.92 ± 0.02	0.88 ± 0.03	0.92 ± 0.03	0.87 ± 0.02

Measurements obtained from ^1^ tail and ^2^ femoral arteries. Data are expressed as mean ± SEM (*n* = 5–10/group). For means within the same row, different letters superscripts [a,b] represent significant differences (*p* < 0.05). An absence of letters indicates no statistical differences. Abbreviations: DBP, diastolic blood pressure; HR, heart rate; HRV, heart rate variability; MAP, mean arterial pressure; MinFV, minimum flow velocity; MFV, mean flow velocity; PFV, peak flow velocity.

**Table 4:** Phase 1: Vascular geometry.

	WKY	SHR		
	Control	Control	Navy Bean	Black Bean
**Aorta**				
Lumen diameter (µm)	1680 ± 33	1710 ± 20	1680 ± 37	1670 ± 21
Lumen CSA (µm^2^)	1970 ± 90	2040 ± 72	1980 ± 86	1990 ± 54
Media thickness (µm)	63.1 ± 1.9 ^b^	93.4 ± 3.3 ^a^	85.9 ± 5.0 ^a^	84.7 ± 2.1 ^a^
Media CSA (µm^2^)	326 ± 12 ^b^	498 ± 24 ^a^	478 ± 27 ^a^	444 ± 19 ^a^
Media:lumen ratio (× 100)	3.76 ± 0.14 ^b^	5.48 ± 0.21 ^a^	5.12 ± 0.32 ^a^	5.08 ± 0.12 ^a^
External diameter (µm)	1810 ± 34	1900 ± 19	1860 ± 38	1840 ± 23
Adventitia thickness (µm)	81.4 ± 9.7	64.8 ± 9.3	62.5 ± 10.9	61.0 ± 4.9
Adventitia CSA (µm^2^)	358 ± 20	302 ± 49	337 ± 51	384 ± 28
**Mesenteric resistance arteries**				
Lumen diameter (µm)	340 ± 8 ^a^	250 ± 7 ^b^	260 ± 13 ^b^	270 ± 11 ^b^
Media thickness (µm)	25.5 ± 1.2 ^b^	34.5 ± 1.4 ^a^	35.5 ± 1.4 ^a^	35.9 ± 1.5 ^a^
Media CSA (µm^2^)	29380 ± 1892	30510 ± 2058	33440 ± 2603	35080 ± 2602
Media:lumen ratio	7.54 ± 0.28 ^b^	13.54 ± 0.41 ^a^	13.71 ± 0.63 ^a^	13.26 ± 0.37 ^a^
External diameter (µm)	390 ± 9 ^a^	320 ± 9 ^b^	330 ± 14 ^b^	340 ± 14 ^b^

Data are expressed as mean ± SEM (*n* = 5–10/group). For means within the same row, different letters superscripts [a,b] represent significant differences (*p* < 0.05). An absence of letters indicates no statistical differences. Abbreviations: CSA, cross-sectional area; SHR, spontaneously hypertensive rat; WKY, Wistar Kyoto.

**Table 5:** Phase 2: Metabolic parameters.

	SHR			
	Baseline	Black Beans for 8 Weeks	Washout for 2 Weeks	Washout for 4 Weeks
**Feed intake (g/day)**	26.8 ± 2.4 ^b^	38.7 ± 2.1 ^a^	44.0 ± 2.3 ^a^	46.0 ± 1.9 ^a^
**Body weight (g)**	301 ± 5 ^c^	370 ± 6 ^b^	389 ± 6 ^a,b^	403 ± 8 ^a^
**Triglycerides (mmol/L)**	0.77 ± 0.09 ^a^	0.51 ± 0.03 ^b^	0.54 ± 0.03 ^b^	0.53 ± 0.04 ^b^
**Total cholesterol (mmol/L)**	1.91 ± 0.10 ^a^	1.60 ± 0.06 ^b^	1.84 ± 0.08 ^a,b^	1.71 ± 0.08 ^a,b^
**HDL-cholesterol (mmol/L)**	1.29 ± 0.09	1.17 ± 0.04	1.36 ± 0.03	1.20 ± 0.05
**LDL-cholesterol (mmol/L)**	0.20 ± 0.07 ^b^	0.37 ± 0.03 ^a,b^	0.44 ± 0.02 ^a^	0.39 ± 0.04 ^a^

Data are expressed as mean ± SEM (*n* = 10/group at baseline and 8 weeks; *n* = 5/group for washout for 2 or 4 weeks). For means within the same row, different letters superscripts [a,b,c] represent significant differences (*p* < 0.05). An absence of letters indicates no statistical differences. Abbreviations: HDL, high-density lipoprotein; LDL, low-density lipoprotein; SHR, spontaneously hypertensive rat.

**Table 6:** Phase 2: Hemodynamic properties and arterial stiffness.

	SHR			
	Baseline	Black Beans for 8 Weeks	Washout for 2 Weeks	Washout for 4 Weeks
**Hemodynamics**				
DBP (mmHg) ^1^	123 ± 4	136 ± 6	141 ± 8	135 ± 12
SBP (mmHg) ^1^	165 ± 5	187 ± 6	188 ± 10	187 ± 11
MAP (mmHg) ^1^	137 ± 4	153 ± 6	156 ± 9	152 ± 12
HR (bpm) ^2^	377 ± 9 ^b^	466 ± 14 ^a^	427 ± 14 ^a^	455 ± 34 ^a^
HRV (bpm) ^2^	161 ± 3 ^a^	139 ± 3 ^b^	150 ± 3 ^b^	143 ± 7 ^b^
**Femoral artery stiffness**				
PFV (cm/sec)	31.8 ± 0.6 ^a^	28.9 ± 1.6 ^a,b^	25.2 ± 0.9 ^b^	27.3 ± 1.7 ^b^
MinFV (cm/sec)	3.5 ± 0.4 ^a^	0.2 ± 0.7 ^b^	1.6 ± 0.5 ^b^	1.5 ± 0.7 ^b^
MFV (cm/sec)	9.7 ± 0.5 ^a^	5.9 ± 0.5 ^b^	7.5 ± 0.6 ^b^	7.4 ± 1.2 ^b^
Pulsatility index	3.0 ± 0.1 ^b^	6.0 ± 1.3 ^a^	3.4 ± 0.3 ^b^	3.8 ± 0.4 ^b^
Resistivity index	0.89 ± 0.01 ^b^	0.99 ± 0.02 ^a^	0.94 ± 0.02 ^a,b^	0.95 ± 0.02 ^a,b^

Measurements obtained from ^1^ tail artery, ^2^ femoral artery. Data are expressed as mean ± SEM (*n* = 10/group at baseline and 8 weeks; *n* = 5/group for washout for 2 or 4 weeks). For means within the same row, different letters superscripts [a,b] represent significant differences (*p* < 0.05). An absence of letters indicates no statistical differences. Abbreviations: DBP, diastolic blood pressure; HR, heart rate; HRV, heart rate variability; MAP, mean arterial pressure; MinFV, minimum flow velocity; MFV, mean flow velocity; PFV, peak flow velocity; SHR, spontaneously hypertensive rats.

**Table 7:** Phase 2: Large-artery geometry^1^.

		SHR	
	Black Beans for 8 Weeks	Washout for 2 Weeks	Washout for 4 Weeks
Lumen diameter (µm)	1670 ± 21	1847 ± 107	1732 ± 31
Lumen CSA (µm^2^)	1990 ± 54	2487 ± 286	2252 ± 119
Media thickness (µm)	84.7 ± 2.1 ^b^	108 ± 8 ^a^	122 ± 15 ^a^
Media CSA (µm)	444 ± 19 ^b^	670 ± 84 ^a^	712 ± 90 ^a^
Media:lumen ratio (× 100)	5.08 ± 0.12 ^b^	5.9 ± 0.3 ^b^	7.1 ± 0.9 ^a^
External diameter (µm)	1840 ± 23	2187 ± 137	2104 ± 54
Adventitia thickness (µm)	61.0 ± 4.9	62 ± 10	64 ± 11
Adventitia CSA (µm^2^)	384 ± 28	396 ± 74	353 ± 55

^1^ Measurements obtained from aorta cross-sections. Data are expressed as mean ± SEM (*n*=4–7/group). For means within the same row, different letters represent significant differences (*p*<0.05). An absence of letters indicates no statistical differences. Abbreviations: CSA, cross-sectional area; SHR, spontaneously hypertensive rat.

## Supplementary Materials

The following are available online at https://www.mdpi.com/2072-6643/12/3/685/s1, Table S1: Proximate composition of beans and experimental diets.
