# Supplementary material for: Regular Black Bean Consumption Is Necessary to Sustain Improvements in Small-Artery Vascular Compliance in the Spontaneously Hypertensive Rat
Source: Nutrients. 2020 Mar 3;12(3):685. doi: 10.3390/nu12030685 (PMC7146454; doi:10.3390/nu12030685)
Supplement: Supplementary file 1 [file nutrients-12-00685-s001.pdf]

Table S1. Proximate composition of beans[1] and experimental diets.

Proximate Analysis (g/100 g DW)	Analytical Method	Control Diet	Navy Bean Diet	Black Bean Diet	Navy Bean Powder	Black Bean Powder
**Crude protein**	AOAC 990.3	16.8	17.8	16.5	24.0	22.8
**Crude fibre**	AOCS Ba6a-05	1.04	3.51	3.04	5.4	4.9
**Non-fibre carbohydrate**	n/a	65.7	64.4	64.0	64.8	66.7
**Fat**	AOCS Am 5-04	6.89	7.04	7.76	2.48	2.18
**Ash**	AOAC 923.03	2.39	3.19	3.25	2.40	2.45
**Energy (kcal/g)**	n/a	3.92	3.92	3.92	3.77	3.77

Determinations based on dry weight of material. Proximate analysis was conducted by Central Testing Labs Ltd. (Winnipeg, MB, Canada). Proximate analyses without an analytical method were determined by calculation. [1]Analysis conducted on lyophilized, milled bean powders. *Abbreviations:* AOAC, Association of Official Analytical Chemists; AOCS, American Oil Chemist Society; DW, dry weight.
